# Traditional Chinese medicine: a promising candidate for the treatment of Alzheimer’s disease

**DOI:** 10.1186/2047-9158-2-6

**Published:** 2013-02-28

**Authors:** Zhi-Kun Sun, Hong-Qi Yang, Sheng-Di Chen

**Affiliations:** 1 Department of Neurology, Henan Provincial People’s Hospital, 450003, Zhengzhou, Henan Province, People’s Republic of China; 2 Department of Neurology and Institute of Neurology, Ruijin Hospital, Shanghai JiaoTong University School of Medicine, 200025, Shanghai, People’s Republic of China

**Keywords:** Alzheimer’s disease, Chinese traditional medicine, Therapeutic approach

## Abstract

Alzheimer’s disease (AD) is an age-related neurodegenerative disorder, characterized clinically by insidious onset of memory and cognition impairment, emergence of psychiatric symptoms and behavioral disorder, and impairment of activities of daily living (ADL). Traditional Chinese medicine (TCM) is practiced in the Chinese health care system for more than 2,000 years. In recent years, scientists have isolated many novel compounds from herbs, some of which improve dementia with fewer side effects than conventional drugs and are regarded as potential anti-AD drugs. In this review, we summarize the latest research progress on TCM showing their possible role of treatment of AD and other demented diseases and possible pharmacological actions.

## Introduction

Alzheimer’s disease (AD) is an age-related neurodegenerative disorder, characterized clinically by insidious onset of memory and cognition impairment, emergence of psychiatric symptoms and behavioral disorder, and loss of activities of daily living (ADL). The histopathological hallmarks of AD include the formation of senile plaques (SP), neurofibrillary tangles (NFTs) and the loss of neurons. With the rapid increase in the aged population in recent years, senile dementia has become one of the world’s important public health issues. AD is the most common type of senile dementia. It is estimated that the prevalence of AD over the age of 85 may be as high as 25 ~ 50%, and AD is increasingly being recognized as one of the most important medical problems in the elderly. It is very important to identify novel and pharmacotherapeutic drugs for AD. Traditional Chinese medicine (TCM) is practiced in the Chinese health care system for more than 2,000 years. Many active pharmacological compounds from Chinese herbal medicines have been identified for the treatment of various diseases. In recent years, scientists have isolated many novel compounds from herbs, some of which improve dementia with fewer side effects than conventional drugs and are regarded as potential anti-AD drugs. In this review, we summarize the latest research progress on Chinese herbal medicines showing their roles of treatment of AD and other demented diseases and possible pharmacological actions.

### Huperzine A

Huperzine A (HupA) is an extract of Huperzia serrata, it has been used for centuries in China to treat fever, swelling, and blood disorders. HupA is a potent, reversible, and selective inhibitor of acetylcholine esterase, exhibiting rapid absorption and penetration into the brain in animal studies, and it has been widely used in China for the treatment of AD. HupA was reported to reduce glutamate-induced toxicity in neurons, possibly through modulation of glutamate-NMDA receptor interaction, or of the passage of Ca2+ through associated ion channels [1]. In addition to the hypothesized reduction in excitotoxicity, other neuroprotective and antioxidant properties have been suggested as well [2]. A recent discovery from Yang et al. verified the co-occurrence of the beneficial effects of HupA on mitochondrial dysfunction and memory deficits in APP/PS1 double transgenic mice, at a time point that AChE was not inhibited [3]. Another study indicates that HupA treatment can significantly ameliorate the loss of dendritic spine density and synaptotagmin levels in the brain of APP/PS1 transgenic mice, and this neuroprotection was associated with reduced amyloid plaque burden and oligomeric beta-amyloid (Abeta) levels in the cortex and hippocampus [4]. HupA can improve triple transgenic mice (3xTg-AD) learning and memory in the Morris water maze and some indicators of emotionality without inducing important adverse effects. Moreover, HupA activate protein kinase C/mitogen-activated protein kinase pathway signalling, alpha-secretases (ADAM 10 and TACE) and increase the fraction of phospho-glycogen synthase kinase 3-beta in 3xTg-AD mice [5]. And it could activate Wnt signaling and reduce amyloidosis in APP/PS1 transgenic mice brain [6].

In a meta-analysis of the efficacy and safety of HupA in AD, The author searched for randomized trials comparing HupA with placebo in the treatment of AD. The primary outcome measures were mini-mental state examination (MMSE) and ADL. Data were extracted from four randomized clinical trials and analyzed using standard meta-analysis and meta-regression methods. Oral administration of HupA for 8–24 weeks (300–500 μg daily) led to significant improvements in MMSE and ADL. Most adverse events were cholinergic in nature and no serious adverse events occurred [7]. However, another study assessed the safety, tolerability, and efficacy of HupA in mild to moderate AD in a multicenter trial in which 210 individuals were randomized to receive placebo (n = 70) or HupA (200 μg BID [n = 70] or 400 μg BID [n = 70]), for at least 16 weeks, with 177 subjects completing the treatment phase. This study provides evidence that HupA 200 mg BID has no demonstrable cognitive effect in patients with mild to moderate AD [8]. A randomized, multicenter trial in China showed that in a 12 weeks treatment period (400 μg/QD), HupA improved 4.6 points in cognition assessed by ADAS-Cog, 2.7 points by MMSE and 2.4 points by ADL in comparison with baseline data. Only mild and transient adverse events (edema of bilateral ankles and insomnia) were observed in 3% of huperzine treated patients. These results indicated its safety and effective in AD treatment [9].

### Gingko biloba

The Ginkgo tree is indigenous to Korea, Japan, China, now it can be found worldwide. Extracts from Ginkgo biloba have been used medicinally in TCM for thousands of years to treat circulatory problems, asthma, vertigo, fatigue, and tinnitus. Ginkgo biloba has been used traditionally in Iran to improve memory loss associated with blood circulation abnormalities. Recently, it has become more widely used in the United States to treat age-related physical and cognitive disorders. The Ginkgo biloba extract EGb 761 has shown favourable effects on cerebral circulation and neuronal cell metabolism, on the muscarinic cholinergic system, and showed antioxidant activity [10]. EGb761 was also neuroprotective against Abeta- and NO-induced toxicity in vitro [11], and could reduce apoptosis both in vitro and in vivo [12]. Using hippocampal neuronal cultures, Shi et al. found EGb761 can block Abeta1-42-induced Ca^2+^ dyshomeostasis mediated by formation of toxic mediators such as H_2_O_2_ and PAF [13].

Treatment with Ginkgo biloba extracts attenuated scopolamine-induced amnesia in rats, enhanced memory retention in young and old rats and improved short-term memory in mice [14]. The clinical efficacy of Ginkgo biloba extracts, including EGb 761, was observed (modest improvements in cognitive function) following administration to AD and non-AD patients in various studies [15,16]. In a 24-week randomised controlled trial, using 240 mg once-daily preparation of Ginkgo biloba extract EGb 761 in 404 outpatients, EGb 761 can improve cognitive functioning, neuropsychiatric symptoms and functional abilities in AD and VaD [17]. However, in Ginkgo Evaluation of Memory (GEM) study, a randomized, double-blind, placebo-controlled clinical trial of 3069 community-dwelling participants aged 72 to 96 years, with a median follow-up of 6.1 years, Snitz et al. reported that the use of G. biloba, 120 mg twice daily, did not result in less cognitive decline in older adults with normal cognition or with mild cognitive impairment [18], Which results has the negative study and it different from the results studied before. We thought the reason is the Participants in that study chosed are the older adults with MCI and not AD or VD. It is apparent that G. biloba can be useful in the treatment of AD symptoms, but further research is necessary to identify appropriate dosing regimens. Moreover, the potential effects of long-term use, interactions with other medicines, and standardization of extracts must be a consideration.

### Curcumin

Curcuma longa is a member of the ginger family indigenous to South and Southeast Asia, where it is grown commercially. Turmeric is derived from the rhizome (root) of the plant, whose most important commercial application is curry. Curcumin was isolated in 1815, initially named diferuloylmethane. Preparations made from Curcuma longa have been used for centuries in ayurvedic medicine to treat a variety of ailments. These may be taken orally for dyspepsia, liver disease, flatulence, urinary tract disease, as a “blood purifier,” or used topically for a variety of skin ailments. In vitro and in vivo evidence has suggested several possible mechanisms of its action relevant to AD, such as, anti-oxidant, anti-inflammatory properties, and a direct effect against Abeta aggregation [19,20]. Several animal studies suggest that this agent may reduce oxidative damage and amyloid pathology in Alzheimer transgenic mice and may modulate amyloid-induced cytopathology or macrophage processing of amyloid [21]. In rat model of sporadic dementia of Alzheimer’s type, curcumin can ameliorate the cognitive deficits and neurodegeneration [22]. In our previous studies, we found that curcumin showed obvious inhibitory effect on the apoptosis induced by Abeta(25–35) [23]. In another report, we also found that curcumin suppressed activated astroglia induced by Abeta and it might act as a PPARgamma agonist to inhibit the inflammation in Abeta-treated astrocytes [24]. Curcumin can inhibit Abeta-induced cytotoxicity in primary prefrontal cortical neurons by MTT and TUNEL assays. Curcumin also corrected Abeta-induced caspase-3 activation, Bcl-2 downregulation and Akt phosphorylation [25]. In a 24-week randomized, double blind, placebo-controlled study, curcumin was generally well-tolerated though three subjects on curcumin withdrew due to gastrointestinal symptoms. But the researchers were unable to demonstrate clinical or biochemical evidence of efficacy of curcumin in AD in this 24-week placebo-controlled trial though preliminary data suggested limited bioavailability of this compound [26]. However, No clinical trials of curcumin in AD patients have been proved to be valid, the reason that curcumin has not been a miracle cure is that it is poorly absorbed in humans, and its use as a treatment for AD is currently under investigation.

### Salidroside

The rhizome of Rhodiola rosea L has been used in East Asia as a tonic and anti-aging agent since ancient times. There has been mounting evidence that the extract from the rhizome of Rhodiola rosea L possesses significant neuroprotective activity and antioxidative effects. Salidroside is one of the major compounds from the water extracts of the rhizome of Rhodiola rosea L. In a previous study, salidroside could significantly inhibite O_2_^−^-or H_2_O_2_-induced neurotoxicity in rat hippocampal neurons. Several in vitro studies shown that salidroside can protect Abeta-induced oxidative damage on rat neuronal PC12 cells and on SH-SY5Y human neuroblastoma cells [27]. In a in vivo study, salidroside can protect hippocampal neurogenesis against streptozotocin-induced neural injury in rat [28]. In our previous studies, we found that salidroside was able to attenuate abnormal processing of amyloid precursor protein by decreased BACE1 protein level and promoted the secretion of sAPPalpha in hypoxic condition in SH-SY5Y cells [29]. However, other study showed that salidroside has no neuroprotective effects on neuronal cell death induced by Abeta in PC12 cells [30]. Further studies are needed to confirm the effect of salidroside in AD treatment.

### Periwinkle-vinca minor (vinpocetine)

Periwinkle has historically been used to treat a wide variety of diseases. It was used as a folk remedy for diabetes in Europe for centuries. In India, juice from the leaves was used to treat wasp stings. In Hawaii, the plant was boiled to make a poultice to stop bleeding. In China, it was used as an astringent, diuretic, and cough remedy. In Central and South America, it was used as a homemade cold remedy to ease lung congestion and inflammation, as well as sore throats. Numerous mechanisms of action have been proposed for vinpocetine; it has been reported to improve cerebral metabolism, increase glucose and oxygen consumption by the brain, and improve brain resistance to hypoxia [31]. A neuroprotective effect has been claimed through blocking voltage-gated sodium channels, modulating neurotransmitter release, and potentiating the effect of adenosine in cytotoxic hypoxia [32]. Vinpocetine has been reported to have a variety of actions that would hypothetically be beneficial in AD. vinpocetine can ameliorate intracerebroventricular streptozotocin induced cognitive dysfunction and oxidative stress [33]. Several double-blind studies have evaluated vinpocetine for the treatment of AD and related conditions [34]. The best of these was a 16-week double-blind placebo-controlled trial of 203 people with mild to moderate dementia, in which vinpocetine significantly benefit the treated group [34,35]. Meta-analysis of older studies of vinpocetine in poorly defined dementia populations concluded that there is insufficient evidence to support its clinical use at this time.

### Centella asiatica L

Centella asiatica leaf is an ancient Ayurvedic remedy, which is used as a revitalizing herb that strengthens nervous function and memory. It is reported to restore youth, memory and longevity [36]. The herb is also taken as a tonic for poor digestion and rheumatism; the latter suggest it may have anti-inflammatory effects. Centella asiatica is also used in TCM for combating physical and mental exhaustion [37]. In mice, an extract of Centella asiatica leaf was sedative, antidepressant and showed cholinomimetic activity, which was blocked by atropine. These results indicate that Centella asiatica may be appropriate to treat symptoms of depression and anxiety in AD, and may also enhance cholinergic activity and thus, cognitive function. An aqueous extract of Centella asiatica leaf improved learning and memory processes in rats, and modulated dopamine, 5-hydroxytryptamine (5-HT) and noradrenaline systems in rat brain in vivo [38]. The triterpene asiatic acid and its derivatives have been shown to protect cortical neurons from glutamate-induced excitotoxicity in vitro [39]. In the brains of APP/PS mice, Centella asiatica extract can impact the amyloid cascade altering amyloid beta pathology and modulating components of the oxidative stress response that has been implicated in the neurodegenerative changes that occur with AD [40]. In an intracerebroventricular streptozotocin model of AD, Centella asiatica extract is effective in preventing the cognitive deficits, as well as the oxidative stress in rats [41]. In another in vivo study, Centella asiatica extract can significantly attenuate intracerebroventricular colchicine-induced cognitive impairment and associated oxidative damage in rat. Further studies are necessary to confirm this to identify any potential relevance in AD treatment.

### Melissa officinalis L

Melissa officinalis (Labiatae) leaf has been used as a medicinal plant for more than 2000 years. In traditional European medicine, Melissa officinalis was used as a calming and strengthening remedy and to treat migraines, melancholia, neuroses and hysteria, and the plant has been acclaimed for promoting long life and for restoring memory [42]. The primary monoterpenes identified in the essential oil of Melissa officinalis include citral (geranial and neral) [43] which is a weak inhibitor of AChE [44]. Melissa officinalis has been the subject of research regarding its potential as sedative and anxiolytic activities that may be appropriate to provide symptomatic relief for behavioural problems such as agitation in AD. Other activities of Melissa officinalis leaf extracts that may be useful for AD therapy include antioxidant effects [45] and binding to muscarinic and nicotinic receptors in vitro [46], which suggests that favourable effects on cholinergic function may occur in AD patients. In some studies, Melissa officinalis can reduce the cognitive deficits and has a good sedative effect in patients with AD [47]. Previous clinical studies showed that the extract of Melissa officinalis reduces laboratory-induced stress [48] and might have benefits in mood improvement [49]. The use of these herbs and formulations should be well tolerated and adverse effects have not yet been reported. Further studies should be conducted to compare the current therapies for AD and the use of these herbal remedies in controlling the symptoms of AD.

### *Polygala tenuifolia* willd

Polygala tenuifolia (Polygalaceae) root is used in TCM as a cardiotonic and cerebrotonic, as a sedative and tranquillizer, and for amnesia, forgetfulness, neuritis, nightmares and insomnia [50]. According to the Chinese Materia Medica, the root is supposed to have a special effect upon the will and mental powers, giving strength of character, improving understanding, strengthening the memory, and increasing physical powers. There have been numerous studies regarding the reputed memory enhancing potential of Polygala tenuifolia root. For example, the traditional Chinese prescription DX-9386, composed of four herbs (Panax ginseng, Polygala tenuifolia, Acorus gramineus and Poria cocos) has shown favourable effects in relation to AD symptoms in several animal models. Tenuifolin inhibited amyloid-beta secretion in vivo and vitro [51]. DX-9386 improved motor activity, reduced lipid peroxidation, ameliorated memory impairment and prolonged the lifespan of senescence accelerated mice and, ameliorated the ethanol- and scopolamine-induced memory impairment in mice [52,53]. Further investigations are required to clarify the contribution of each of the four herbs in DX-9386 to the observed pharmacological activities. GEPT, consisting of extracts from ginseng, epimedium, polygala, and tuber curcumae [54], has shown valuable for the treatment of AD. In the brain of APPV717I transgenic mice, GEPT can reduce the level of Abeta via the inhibition of γ-secretase (presenilin-1) and the promotion of insulindegrading enzyme and neprilysin [55]. It can also increases synaptophysin expression in APPV717I transgenic mice. [56]. Kami-utan-to (KUT), a prescription containing 13 herbs including Polygala tenuifolia root, is used in traditional Japanese medicine to treat psychoneurological diseases. KUT dose-dependently upregulated choline acetyltransferase (ChAT) activity and increased nerve growth factor (NGF) secretion in vitro, and improved passive avoidance behaviour and induced ChAT activity in the cerebral cortex of aged rats, and in scopolamine-induced memory impaired rats in vivo [44,57]. The effects on ChAT activity and NGF secretion in vitro were not as pronounced when treated with KUT in the absence of Polygala tenuifolia root, but Polygala tenuifolia root extract alone did upregulate ChAT activity and increase NGF secretion in vitro. These results suggest that Polygala tenuifolia root, particularly the cinnamic acid derivatives, significantly contribute to the pharmacological activities of KUT, and may explain the reputed beneficial effects of KUT in Japanese medicine.

### *Salvia miltiorrhiza* bung

Throughout history Salvia miltiorrhiza (Labiatae) has been used for the treatment of a variety of medical conditions. The dried root of Salvia miltiorrhiza was used in folk medicine for the management of blood disorders. It is prescribed in TCM to stabilize the heart and calm nerves. Official indications for the root include treatment of blood circulation disorders, insomnia, neurasthenia and alleviation of inflammation [58]. Salvia miltiorrhiza root may inhibit neuronal cell death by inhibition of presynaptic glutamate release, and nitric oxide (NO) formation. Other investigations indicate Salvia miltiorrhiza root may modify ischaemic cell changes by modulating somatostatin, a CNS neuropeptide that has been implicated in learning and memory [59]. The anti-oxidant effects of Salvia miltiorrhiza root have been studied and several compounds have been identified with significant antioxidant activity. Such antioxidant compounds may be useful in AD therapy. Salvia leriifolia Benth (Lamiaceae) extract demonstrates antioxidant properties and cholinesterase inhibitory activity [60]. Cryptotanshinone, a compound from Salvia miltiorrhiza, modulates amyloid precursor protein metabolism and attenuates Abeta deposition through upregulating alpha-secretase in vivo and in vitro [61]. Tanshinones isolated from Salvia miltiorrhiza root have demonstrated antiinflammatory activity in mice and were active against 5-LOX in porcine leukocytes, but were not as active as the crude extracts. These activities need to be further investigated for confirmation, but could be relevant in AD therapy.

### *Withania somnifera* (L.) Dun

Withania somnifera (Solanaceae) root (ashwagandha) is one of the most highly regarded herbs in Ayurvedic medicine and its use dates back almost 4000 years. It is classed among the rejuvenative tonics (‘Rasayanas’). The herb is also traditionally used to treat inflammatory conditions, such as arthritis. Nicotine is reported to be present in Withania somnifera root. The presence of nicotine may explain the reputed activity of Withania somnifera in Ayurvedic medicine, considering that nicotine has been associated with cognitive enhancement and protection against AD development [62,63].There have been numerous studies regarding the cognitive enhancing activities of Withania somnifera. For example some steroidal derivatives that have been isolated from Withania somnifera root are the sitoindosides IX and X, which augmented learning acquisition and memory in both young and old rats. The extract containing the sitoindosides VII–X and withaferin A also reversed the ibotenic acid-induced cognitive deficit and reversed the reduction in cholinergic markers (e.g. ACh, ChAT) in rats [64]. These observations indicate that the sitoindosides VII–X and withaferin A could have potential in AD therapy. A root preparation of Withania somnifera has also been shown to significantly reduce the number of degenerating cells in the hippocampal region of stressed rats, which indicates the root extract may have potential neuroprotective effects in neurodegenerative disorders. Withania somnifera root, leaves and some constituents are also reported to have antioxidant and anti-inflammatory activities, inhibition of human acetyl cholinesterase [65] and protect the PC-12 cells from Abeta induced cell damage [66,67], which may also be relevant in AD therapy [68].

## Conclusions

The treatment of AD remains a challenge in the modern medicine because of the pathogenesis of AD is a complex process involving both genetic and environmental factors; therefore development of effective disease-modifying drugs is proving to be a difficult task. These traditional Chinese medicines are regarded as new and promising sources of potential anti-AD drugs. These encouraging preclinical and clinical trials suggest that TCM is a promising candidate for the treatment of AD.

## Abbreviations

AD: Alzheimer’s disease; TCM: Traditional Chinese medicine; Abeta: Beta amyloid; APP: Amyloid precursor; HupA: Huperzine A;3xTg-AD: Triple transgenic mice; MMSE: Mini-mental state examination; ADL: Activities of daily living scale; VaD: Vascular dementia; GEM: Ginkgo evaluation of memory; 5-HT: 5-Hydroxytryptamine; ChAT: Choline acetyltransferase; NGF: Nerve growth factor; NO: Nitric oxide.

## Competing interest

The authors declare that they have no competing interests.

## Authors’ contributions

Z-KS and H-QY made equal contributions to conception and design, acquisition of data, and in drafting the manuscript. S-DC was the general supervision of the research group, acquisition of funding, and involved in revising it critically for important intellectual content. All authors read and approved the final manuscript.
